# miR-4666-3p and miR-329 Synergistically Suppress the Stemness of Colorectal Cancer Cells via Targeting TGF-β/Smad Pathway

**DOI:** 10.3389/fonc.2019.01251

**Published:** 2019-11-19

**Authors:** Jun Ye, Jiacai Lei, Qingqing Fang, Yimin Shen, Wenjie Xia, Xiaoge Hu, Qiuran Xu, Hongjun Yuan, Jian Huang, Chao Ni

**Affiliations:** ^1^Key Laboratory of Tumor Microenvironment and Immune Therapy, The Second Affiliated Hospital of Zhejiang University School of Medicine, Hangzhou, China; ^2^Department of Gastroenterology, Hangzhou Dajiangdong Hospital, Hangzhou, China; ^3^Key Laboratory of Tumor Molecular Diagnosis and Individualized Medicine of Zhejiang Province, Zhejiang Provincial People's Hospital, People's Hospital of Hangzhou Medical College, Hangzhou, China; ^4^Department of Endocrinology, The Second Affiliated Hospital of Zhejiang University School of Medicine, Hangzhou, China; ^5^Department of General Surgery, Zhejiang Provincial People's Hospital, People's Hospital of Hangzhou Medical College, Hangzhou, China; ^6^Department of Breast Surgery, The Second Affiliated Hospital of Zhejiang University School of Medicine, Hangzhou, China

**Keywords:** colorectal cancer, miR-4666-3p, miR-329, cancer stem cells, TGF-β/smad

## Abstract

Quiescent caner stem cells are identified as a subpopulation of colon cancer cells in dormant state and possess strong stem-cell like characteristics. Previously, we have identified this subpopulation in colorectal cancer (quiescent colon cancer stem cells, QCCSCs), and find QCCSCs are sensitive to the apoptotic effect of IFN-γ, which is attributed to their high IFN-γR expression levels. Microarray and bioinformatic analysis indicate miR-4666-3p is low expressed in QCCSCs and target IFN-γR1/2, which is proved by luciferase assay and western-blot. Furthermore, we find miR-4666-3p could also target TGF-βR1 to block the activation of TGF-β1/Smad pathway, therefore function as a tumor suppressor gene to inhibit the stemness of colon cancer cells. Besides, compared with QCCSCs, we find the TGF-β1 expression also decreased with the weakening of stemness properties. In terms of mechanism, our result reveal TGF-β1 is the target gene of miR-329, which is also high expressed in non-QCCSCs. Thereafter, we perform gain- and loss- function experiments to confirm the synergistic effect between miR-4666-3p and miR-329 in blocking the activation of TGF-β/Smad pathway. Finally, we evaluate the expression of both miR-4666-3p and miR-329 in 73 tumor specimens and paired normal tissue, and find both two miRNAs are related to unfavorable prognosis and advanced tumor stage in colorectal cancer. Our study revealed a novel epigenetic regulation mechanism in colon cancer stem cells, which could be exploited as a novel therapeutic strategy for cancer treatment.

## Introduction

The existence of quiescent cancer stem cells (QCSCs) has been reported in various solid tumors and QCSCs are defined as a distinct subset of cells in quiescent status or undergoing very slow proliferation that possess a strong capacity for tumorigenesis and a resistance to cytotoxic drugs (1). Previously, we identified a population of quiescent colon cancer cells with strong stemness by PKH staining and a serum-free medium cultivation assay (quiescent colon cancer stem cells, QCCSCs) and reported that QCCSCs are resistant to chemotherapy but sensitive to the apoptotic effect of IFN-γ, which is caused by their high expression of IFN-γ receptors (IFNGRs) (2). Herein, we want to illuminate the mechanisms underlying the different expression patterns of QCCSCs and non-QCCSCs.

Cancer is composed of many genetically distinct subclones that are produced by both intrinsic and therapeutic effects. However, several studies have found that differences in biological behaviors among cancer cells cannot be ascribed to genetic mutations alone. A more in-depth understanding of CSC (cancer stem cell) dynamics has revealed that cells in many tumors exhibit phenotypic plasticity, suggesting a transition between the CSC and non-CSC states. Iliopoulos et al. reported a mutual transition between the CD44^high^ CSC phenotype and the CD44^low^ non-CSC phenotype (3). Roesch et al. described the presence of a population of slow-cycling stem-like melanoma cells, which were characterized by high expression of JARID1B (Jumonji/AT-rich interactive domain-containing protein 1B), but the non-stem-like cells with low expression level of JARID1B could turn on high levels of the protein through epigenetic modulation (4). Based on the observation that some isolated populations could generate other populations to restore equilibrium, the transition from CSCs to non-CSCs must involve epigenetic changes.

Increasing evidence has demonstrated that microRNAs play important roles in maintaining the characteristics of CSCs, such as self-renewal, invasiveness and chemoresistance, and miRNAs could also be used as therapeutic targets or prognostic markers. A previous review indicated that approximately 164 miRNAs were altered in colon cancer. Some play pro-tumorigenic roles, such as miRNA-17, which induces epithelial-mesenchymal transition and promotes the formation of a stem cell-like population in colon cancer cells (5), and some act as tumor suppressive genes, such as miRNA-93, which suppresses the proliferation and tumorigenesis of human colon cancer stem cells (6). Besides, these microRNAs are revealed as important regulators of some key signaling pathway in maintaining stemness of CSCs. Among them, TGF-β/Smad pathway is acknowledged holding a key position in cancer stem cells, and reported there is a dual directional regulation mechanism with microRNAs: TGF-β could induce epithelial-to-mesenchymal transition (EMT) and stemness in pancreatic ductal adenocarcinoma via induces miR-100 and miR-125b but blocks let-7a (7); meanwhile, TGF-β pathway could also be regulated by microRNAs: TrkC-miR2 and miR-140 inhibit TGF-β pathway via direct targeting smad3 (8, 9).

Here, we report that miR-4666-3p and miR-329 are both expressed at low levels in QCCSCs, and both of their expression levels increased with cell division. Furthermore, we found that these two miRNAs work in concert to suppress tumorigenesis and stemness via the TGF-β/Smad pathway, which has been shown to play vital roles in stemness maintenance in colon CSCs (CCSCs). Understanding the mechanisms of how microRNAs regulate the stemness of colon cancer stem cells establishes a strong rationale for their application as prognostic markers and therapeutic markers.

## Materials and Methods

### Colon Cancer Cell Preparation and Cell Lines

P1 cancer cells were established from liver metastasis lesions of colon cancer (10) and were cultured in RPMI 1640 (Invitrogen, Grand Island, NY) medium containing 10% FBS. The colon cancer cell line SW480 was maintained in Leibovitz L-15 (Invitrogen, Grand Island, NY) medium with 10% FBS; 293T cells were maintained in DMEM (Invitrogen, Grand Island, NY), supplement with NaHCO3 1.5 g/L, medium with 10% FBS. The SW480 and 293T cell line was obtained from the Cell Bank of Chinese Academy of Science, Shanghai. Tumor samples were collected from patients undergoing surgery at the Department of Zhejiang Provincial People's Hospital.

This study was approved by the ethics committees of the Zhejiang Provincial People's Hospital (KY2014-AF-09). Informed consent was obtained from all of the enrolled subjects, and the study was performed in full compliance with all principles of the Helsinki Declaration.

### Sphere Formation and FACS Procedures

For isolating PKH^hi^ cells, colon cancer cells were suspended in Diluent C (~1 × 10^7^ cells/ml) and then labeled with PKH26/67 (2 × 10^−6^ M, 5 min, Sigma-Aldrich, USA). The staining efficiency was confirmed with FCM (flow cytometry). Thereafter, 1,000 cells/well were cultured in serum-free medium with F-12, basic fibroblast growth factor, N2 and heparin, and the cells were cultured for 2 weeks in ultralow attachment six-well plates. PKH^hi^ subpopulation cells were defined as previously reported and isolated by FACS (Fluorescence Activated Cell Sorting) (2).

In the series sphere formation assay, single cells were obtained from spheroids digested with Accutase (Invitrogen, Grand Island, NY) (37°C, 5 min) and filtered through a 40 μm strainer and then seeded into ultralow attachment plates. The ratio of spheroid formation was calculated according to the primary seed cell numbers. The cells were seeded into ultralow attachment plates at a density of 1,000 cells per well.

### Flow Cytometry and Western Blot Assay

Antibodies against IFN-γR1 (BB1E2, 1:1000), IFN-γR2 (GTX81601, 1:1000), TGF-βR1 (E161, 1:1000), TGF-β1 (7F6, 1:1000), Smad2/3 (GTX111123, 1:1000), p-smad2 (GTX133614, 1:1000), and p-smad3 (GT1017, 1:1000) were obtained from GeneTex (Irvine, USA).

Flow cytometry was performed according to the manufacturer's protocols. Briefly, to detect the PKH26 fluorescence intensity and TGF-β1 expression (7F6, 1:400, GeneTex, Irvine, USA) in PKHhi/low/neg cells, spheroids were isolated into single cell suspension with Accumax (Invitrogen, San Diego, USA), then permeabilize cells with Intracellular Staining Permeabilization Wash Buffer (Biolegend, USA) and stained with antibody.

Western blot analysis was performed according to the previously reported (11). Briefly, cells were lysed in a RIPA buffer (Cell Signaling Technology, Danvers, MA, USA) and centrifuged. Protein products were separated by 10% SDS-PAGE electrophoresis and transferred to nitrocellulose membranes (Millipore, Billerica, MA, USA). Membranes were blocked with 1.5% BSA in Tris-buffered saline buffer with 0.1% Tween-20 (TBST) for 2 h and incubated with primary antibodies overnight at 4°C. Then membranes were incubated with the corresponding horseradish peroxidase (HRP)-conjugated secondary antibodies for 2 h at room temperature. Detection was performed using the electrochemiluminescence (ECL) kit (Pierce, Rockford, IL, USA).

### Quantitative Reverse-Transcription Polymerase Chain Reaction (qRT-PCR)

Total RNA from colon rectal cancer (CRC) cells was obtained with Trizol reagent (Invitrogen), and total RNA from paraffin-embedded tissues was isolated by the RecoverAll™ Total Nucleic Acid Isolation Kit (Applied Biosystems). Taqman assays (KLF4, OCT4, NANOG, SOX2, miR-4666-3p, and miR-329) were purchased from Applied Biosystems (Life Technologies Co.), performed with the TaqMan Universal PCR Master Mix and analyzed with an ABI Step One Plus System (Applied Biosystems). Because of the extremely low amount of total RNA extracted, especially from the PKH^hi^ cells, we used the Taqman MicroRNA Cells to CT™ Kit (Life Technologies Co.). All samples were run in triplicate, and microRNA expression levels in each sample were normalized to U6 levels.

### miRNA Expression Microarray Analysis

Total RNA was isolated from QCCSCs (PKH^hi^ cells) and non-QCCSCs (PKH^low^ and PKH^neg^ cells) with Trizol reagent. The quantity of RNA was verified with a Nanodrop spectrophotometer (Thermo Fisher Scientific, Worcester, MA, USA). The miRNA expression profile of each sample was assessed with a miRNA microarray produced by JOIN GENOME (Hangzhou, China).

### Luciferase Assay

The pmirGLO Dual-Luciferase miRNA Target Expression vectors (Promega) contained the TGFBR1 mRNA 3′UTR with the miR-4666-3p target site or a mutated miR-4666-3p target site and the TGFβ1 mRNA 3′UTR with the miR-329 target site or a mutated miR-329 target site. The pRL-TK plasmids (Promega) were cotransfected into 293T cells with the negative control mimic (5′-UUCUCCGAACGUGUCACGUTT-3′), miR-4666-3p mimic (5′- CAUACAAUCUGACAUGUAUUU-3′), anti-miR-4666-3p mimic (5′- AAAUACAUGUCAGAUUGUAUG-3′), miR-329 mimic (5′- AACACACCUGGUUAACCUCUUU-3′), or anti-miR-329 mimic (5′-AAAGAGGUUAACCAGGUGUGUGUU-3′) (GenePharma Tech, Shanghai, China) using Lipofectamine 2000 (Invitrogen). The ratio of firefly to Renilla luciferase activity was determined with a luminometer using the Dual Luciferase Reporter Assay System (Promega) 48 h after transfection.

### Immunofluorescence Assay

Cells were fixed with 4% paraformaldehyde for 20 min, blocked in 5% normal goat serum and incubated with a primary antibody against TGFBR1 (1:100, E161, Genetex, Irvine, USA), Smad2 (1:500, ab40855, Abcam, USA), or Smad3 (1:500, ab40854, Abcam, USA) (4°C, overnight), followed by Dylight488-conjugated goat anti-rabbit antibody (1:100, ab96983, Abcam, USA) incubation (37°C, 1 h). After immunolabeling, the cells were washed with PBS, stained with DAPI (Sigma, St. Louis, MO, USA) and then viewed under a laser confocal microscope (LCM, Leica SP-5, Germany).

### *In vivo* Tumorigenesis Assay

Six-week-old nude mice were purchased from Shanghai Institutes for Biological Sciences and bred in specific pathogen-free conditions at the Laboratory Animal Research Center of Zhejiang Medical Academy with the permission of the local animal care and ethics committee.

The cells were suspended in 100 μl medium composed of DMEM F12 and Matrigel (BD Pharmingen, San Diego, CA, USA) and then injected subcutaneously into the dorsal side of nude mice to assess their ability to generate tumor xenografts. The volume of the tumors was measured weekly and calculated as length × width × height/2.

### Other Methods

Other related methods, including IC50 determination, ELISA (enzyme-linked immunosorbent assays), plasmid construction, and the establishment of miR-4666-3p and miR-329 stable knockdown and overexpression cell lines are described in Supplementary Material.

### Statistical Analysis

All data are shown as one typical result from 3 independent experiments in similar conditions or as the mean ± SD of 3 independent experiments. Prism 7 software was used to generate graphs and to perform statistical analysis. The RT-PCR results from clinical samples (**Figures 6A,B**) were analyzed with a two-tailed paired Student's *t*-test, and the other results were analyzed with a two-tailed unpaired Student's *t*-test. *P* < 0.05 indicated statistical significance.

## Results

### miR-4666-3p Was Expressed at Low Levels in PKH^hi^ Cells and Targeted IFN-γR1/2

**Figure 1:**
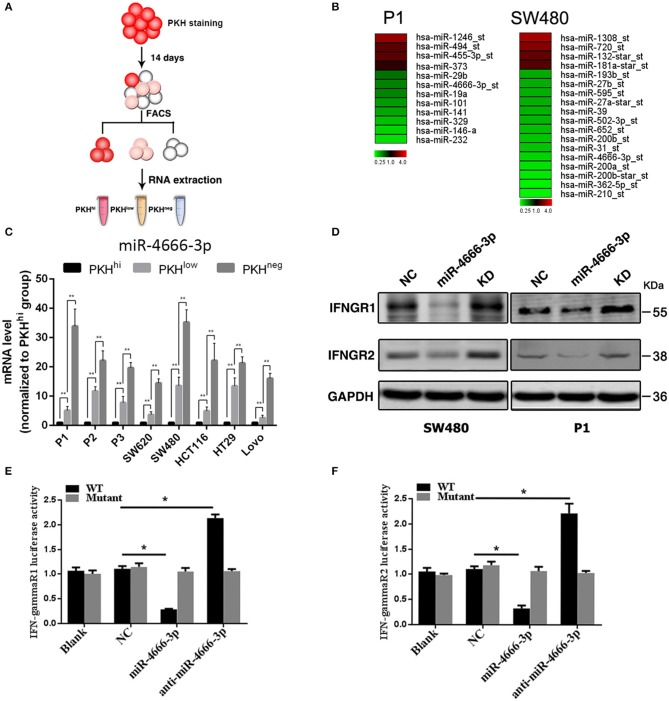
Low expression of miR-4666-3p in the PKH^hi^ subpopulation and miR-4666-3p's effect on its target IFN-γR1/2. **(A)** Schematic model of sorting PKHhi/low/neg subpopulations; **(B)** Differentially expressed miRNA in PKH^hi^ and the rest of the cell population. Red denotes high and green denotes low levels of expression; **(C)** The expression level of miR-4666-3p in different subpopulations of different colon cancer cell lines; **(D)** CRC cells were transfected with the NC, miR-4666-3p or anti-miR-4666-3p mimics, and the expression of IFN-γR1 and IFN-γR2 was detected by Western blotting, KD, knockdown; NC, negative control; **(E,F)** 293T cells were co-transfected with empty pmirGLO Dual-Luciferase reporter plasmids or IFN-γR1 and IFN-γR2 3'UTR firefly luciferase reporter plasmids, pRL-TK-luciferase plasmids, and the miR-4666-3p or anti-miR-4666-3p mimics. After 48 h, firefly luciferase activity was measured and normalized to that of Renilla luciferase. The data are shown as one typical result from three independent experiments with similar results or as the mean ± SD of three independent experiments. *p* < 0.05; *p* < 0.01. WT, wild type. *P < 0.05.

In our previous work, we found that a small subset of slow-cycling cells (PKH^hi^ cells, Figure 1A, Figure S1) exhibited strong cancer stem cell-like features, including high stemness gene expression, great ability of spheroid and xenografts formation and resistance to chemotherapy, therefore, we considered PKH^hi^ cells to be colon cancer stem cells (CCSCs). Meanwhile, we found PKH^hi^ cells were sensitive to IFN-γ-induced apoptosis, which was attributed to their higher IFN-γR1/2 expression level (2). To explore whether microRNAs regulate the IFN-γ receptor levels in PKH^hi^ cells, we performed a microarray analysis to compare the expression patterns of IFNGRs between PKH^hi^ and the remaining cells. The results revealed four upregulated and eight downregulated microRNAs in the PKH^hi^ population of P1 cells and four upregulated and fourteen downregulated microRNAs in the PKH^hi^ population of SW480 cells (Fold change ≥ 2, Figure 1B). Through the comparison, we found that miR-4666-3p was downregulated in the PKH^hi^ population of both cell lines.

The target gene prediction by the Targetscan database (www.targetscan.org) showed that the IFN-γ receptor may be regulated by miR-4666-3p. RT-PCR confirmed that miR-4666-3p was expressed at low levels in PKH^hi^ cells from various colon cancer cell lines, and it is interesting that the expression level gradually increased as the fluorescence intensity decreased (Figure 1C). We further identified both IFN-γR1 and IFN-γR2 as functional downstream targets of miR-4666-3p in SW480 and P1 cells. The protein levels of the IFNGRs were also influenced by transfection of a miR-4666-3p mimic or an anti-miR-4666-3p mimic (Figure 1D). In a dual luciferase reporter assay, we found that the activity of luciferase reporters containing the IFN-γR1 and IFN-γR2 3'UTRs decreased by 70 and 60%, respectively, upon co-transfected with the miR-4666-3p mimic, but both almost doubled upon co-transfected with the anti-miR-4666-3p mimic. Moreover, no change was identified upon co-transfected of the mutant reporter plasmid with either the miR-4666-3p or anti-miR-4666-3p mimic (Figures 1E,F). Finally, we assessed whether miR-4666-3p could regulate the IC50 of IFN-γ and oxaliplatin in colon cancer cell lines. The MTS assay showed that the miR-4666-3p mimic strongly increased the IC50 of IFN-γ in P1 and SW480 cells, while the anti-miR-4666-3p mimic slightly decreased the cell lines' sensitivities to IFN-γ (Figure S2A). In addition, we found that the IC50 of oxaliplatin was significantly increased by transfection of the anti-miR-4666-3p mimic and significantly decreased by transfection of the miR-4666-3p mimic (Figure S2B), which indicated that miR-4666-3p may function as a tumor suppressive gene in colon cancer cells.

### miR-4666-3p Suppresses the Stemness of Colon Cancer Stem Cells

**Figure 2:**
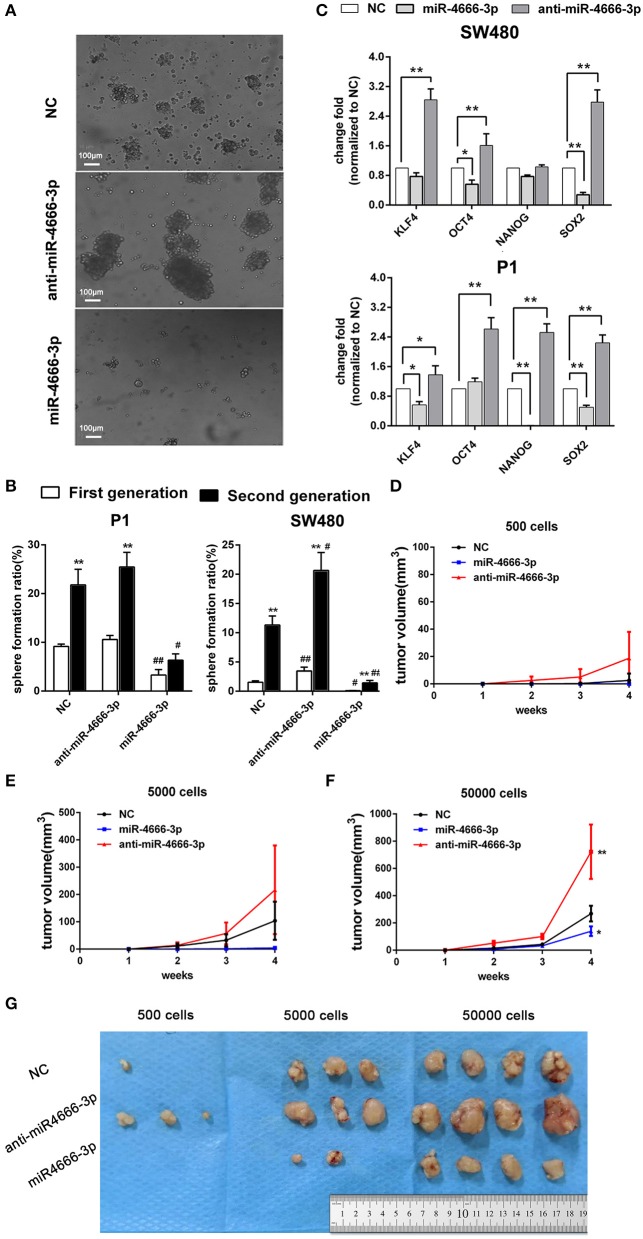
High expression of miR-4666-3p suppresses the stemness of CRC cells. **(A)** Representative images of sphere formation with control, miR-4666-3p mimic and anti-miR-4666-3p mimic transfected P1 cells; **(B)** Serial sphere formation with control, miR-4666-3p mimic and anti-miR-4666-3p mimic transfected CRC cells (* vs. first generation; # vs. NC group, scale bar = 100 μm); **(C)** The expression of stemness genes was assessed by qPCR in control, miR-4666-3p mimic and anti-miR-4666-3p mimic transfected CRC cells; **(D–F)** Comparison of *in vivo* tumorigenesis with the indicated numbers of cells (P1 cells); **(G)** Results from a tumorigenesis assay showing representative images of xenograft tumors in nude mice injected subcutaneously with the indicated numbers of cells (P1 cells). The data are shown as one typical result from three independent experiments with similar results or as the mean ± SD of 3 independent experiments. *,^#^*P* < 0.05, **,^##^*P* < 0.01.

Because the above data demonstrated that miR-4666-3p was expressed at low levels in PKH^hi^ cells and acted as a tumor suppressor gene in CRC, we established both miR-4666-3p- and anti-miR-4666-3p-expressing stable cell lines to study miR-4666-3p's biological function by determining sphere formation *in vitro* and tumorigenesis *in vivo*. We first performed a series sphere formation assay in serum-free medium, and the results revealed that spheroid formation was significantly and inversely related to miR-4666-3p expression in both SW480 and P1 cells (Figures 2A,B). Thereafter, we determined if the expression of four stemness-related genes, OCT4, Nanog, BMI1, and SOX2, were affected by miR-4666-3p levels. Although not all the genes had their expression substantially changed by the miR-4666-3p level, the results showed that the expression of almost all of these genes was dramatically decreased in miR-4666-3p-overexpressing cells and increased in the anti-miR-4666-3p stable cell lines (Figure 2C). Thereafter, we evaluated if miR-4666-3p exert stemness suppressive function via targeting IFN-γ receptors. However, neither change the expression level of IFN-γR1 or IFN-γR2 could affect the spheroid formation ability or stemness gene expression *in vitro* (Figures S3A, B).

In the tumorigenicity assay, the rate of tumor growth was also markedly reduced by miR-4666-3p overexpression in the 500, 5,000, or 50,000 cell groups (Figures 2D–F); besides, P1 cells with low miR-4666-3p expression revealed a much stronger tumor-initiating capacity than P1 cells overexpressing miR-4666-3p; only 500 low-expression P1 cells were needed to form xenografts in nude mice (3/4), while the threshold for xenograft formation in the control (3/4) and miR-4666-3p overexpression groups was ~5,000 cells (2/4) (Figure 2G). These data strongly suggested that miR-4666-3p suppresses tumor-initiating capacity and stemness in colon cancer cells.

### TGF-β Receptor 1 Is a Novel Target of miR-4666-3p

**Figure 3:**
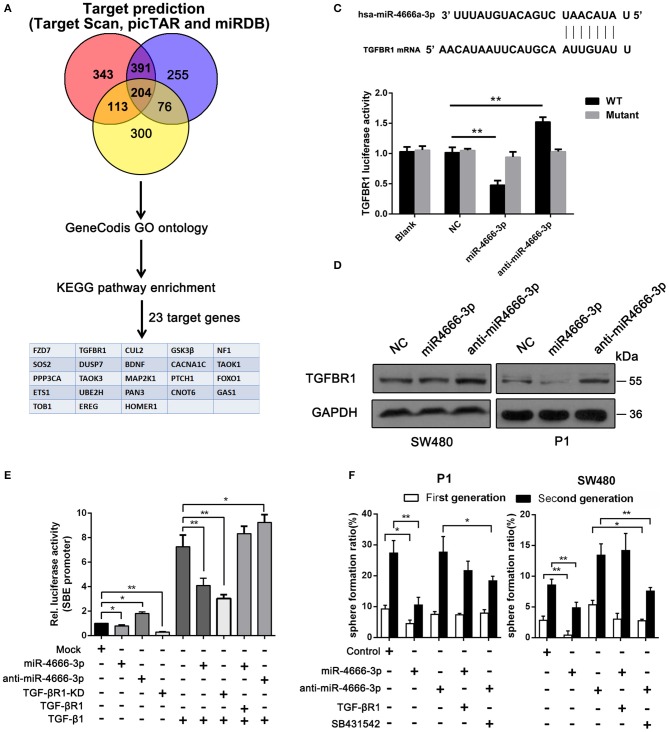
miR-4666-3p targets TGFBR1 in CRC cells. **(A)** Bioinformatics analysis of potential targets of miR-4666-3p; **(B)** TGFBR1 is predicted to be a novel target of miR-4666-3p; **(C)** 293T cells were cotransfected with empty pmirGLO Dual-Luciferase reporter plasmids or TGFBR1 3′UTR firefly luciferase reporter plasmids and pRL-TK-luciferase plasmids, together with the miR-329 or anti-miR-4666-3p mimic. After 48 h, firefly luciferase activity was measured and normalized to that of Renilla luciferase; **(D)** CRC cells were transfected with the NC, miR-4666-3p or anti-miR-4666-3p mimics, and expression of TGFBR1 was detected by Western blotting; **(E)** The activity of the TGF-β/Smad pathway was measured by the SBE luciferase reporter system in colon cancer cells (presented as P1 cells) with distinct treatment combination; **(F)** Serial sphere formation with different cells (control, miR-4666-3p, anti-miR-4666-3p stable expression and both miR-4666-3p and TGF-βR1 stable expression cells). The data are shown as one typical result from three independent experiments with similar results or as the mean ± SD of three independent experiments. *P < 0.05, **P < 0.01.

Because miRNAs usually act as mediators that silence genes, we performed a bioinformatic analysis of the target genes of miR-4666-3p and attempted to elucidate the underlying mechanism of how miR-4666-3p suppresses tumor growth and stemness. First, we analyzed target genes of miR-4666-3p via TargetScan (www.targetscan.org), picTar (https://pictar.mdc-berlin.de/) and miRDB (www.mirdb.org), and 204 genes were found to be predicted by all three databases. Next, we functionally annotated these genes with GeneCodis and then subjected them to further enrichment analysis of cell signaling pathways using the Kyoto Encyclopedia of Genes and Genomes (KEGG) pathway database (www.genome.jp/kegg/). Using this approach (Figure 3A), miR-4666-3p was predicted to target various cancer-related signaling pathways, including the TGF-β, mitogen-activated protein kinase (MAPK) and insulin signaling pathways, which comprised 33 target genes. The KEGG result and network diagram between these genes and miR-4666-3p are presented in Figure S4. Finally, we focused on TGF-βR1 because of its crucial function in the TGF-β pathway, which is one of the most common canonical signaling pathways to promote stemness and EMT in CSCs (12–14).

Next, we identified TGF-βR1 as a functional downstream target of miR-4666-3p. The 3‘UTR of TGF-βR1 contained highly conserved miR-4666-3p binding sites (Figure 3B), which was proven in a luciferase reporter assay. Our results showed that the activity of a luciferase reporter containing the TGF-βR1 3'UTR decreased by 60% upon co-transfected with the miR-4666-3p mimic but increased by 50% upon co-transfected with the anti-miR-4666-3p mimic, and a negative result was found when the mutant reporter plasmid was co-transfected with either the miR-4666-3p mimic or anti-miR-4666-3p mimic (Figure 3C). Western blotting also confirmed that TGF-βR1 protein expression changed with miR-4666-3p overexpression or downregulation (Figure 3D).

### miR-4666-3p Inhibit the TGF-β Pathway and Stemness via Targeting TGF-βR1

Afterwards, we evaluated whether TGF-βR1 affected the stemness of CCSCs. Using a series spheroid formation assay, we found that the knockdown of TGF-βR1 suppressed sphere formation and stem gene expression, while overexpression of TGF-βR1 potentiated sphere formation and stemness gene expression (Figures S5A, B). TGF-βR1 is a key upstream part of activating TGF-β pathway, herein we assessed whether miR-4666-3p could suppress canonical TGF-β/Smad pathway with luciferase reporter gene assays using pGL3-SBE4-Luc plasmids, which report binding to SBE. The result revealed inhibition of TGF-β pathway with TGF-βR1 knockdown or selective TGF-βRIinhibitor (SB-431542, 10 μM) markedly decrease the luciferase activity and sphere formation ability (Figures 3E,F), meanwhile, overexpression of miR-4666-3p significantly inhibit the activity of TGF-β/Smad pathway induced by supplement of TGF-β1 (Figure 3E). Then we constructed TGF-βR1 overexpressing plasmid (without its 3'UTR) and performed rescue experiment. The cells were co-transfected with miR-4666-3p and TGF-βR1 plasmid, in parallel with control group. The restore of TGF-βR1 expression was confirmed with qRT-PCR (Figure S5C) and western-blot (Figure S5D). The re-expression of TGF-βR1 markedly restore the sphere formation ability (Figure 3F) and activity of TGF-β/Smad pathway induced by TGF-β1 (Figure 3E).

### miR-329 Was Expressed at Low Levels in PKH^hi^ Cells and Suppress TGF-β1 Secretion

**Figure 4:**
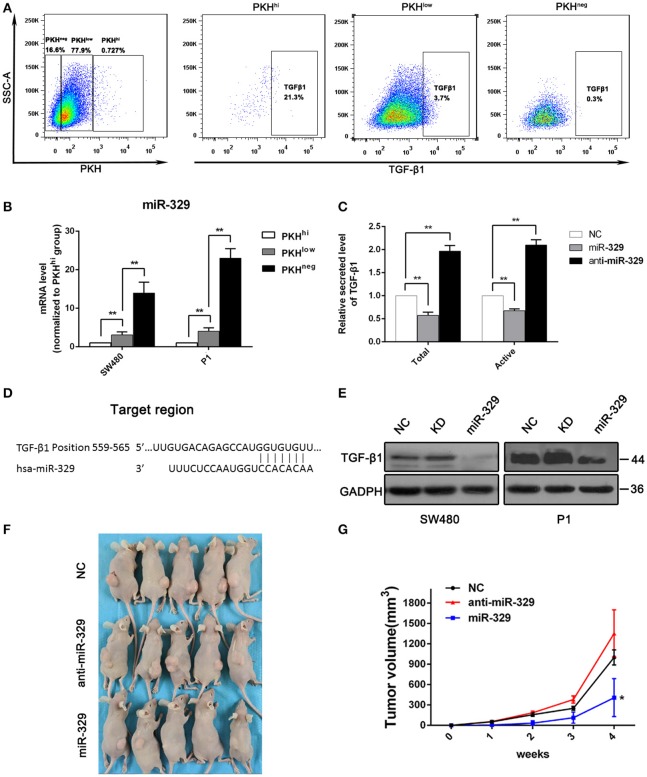
miR-329 is low expressed in PKH^hi^ cells and targets TGFβ1. **(A)** Flow cytometry assessed the expression of TGF-β1 in PKH^hi^, PKH^low^, and PKH^neg^ subpopulations; **(B)** miR-329 expression in PKH^hi^, PKH^low^, and PKH^neg^ subpopulations assessed by qPCR; **(C)** colon cancer cells were transfected with the NC, miR-329 or anti-miR-329 mimic, and total and active TGF-β1 levels in culture medium were detected by ELISA, data was presented with P1 cells; **(D)** TGFβ1 is predicted to be a novel target of miR-329; **(E)** CRC cells were transfected with the NC, miR-329 or anti-miR-329 mimic and expression of TGFβ1 was detected by Western blotting; **(F)** Results from a tumorigenesis assay show representative images of xenograft tumors in nude mice injected subcutaneously with 1 × 10^4^ P1 cells/mice; **(G)** Comparison of tumor growth in nude mice injected with 1 × 10^4^ P1 cells/mice. The data are shown as one typical result from three independent experiments with similar results or as the mean ± SD of three independent experiments. *P < 0.05, **P < 0.01.

The above result indicate TGF-β/Smads pathway was activated with miR-4666-3p downregulation in colon cancer cells, and it has been reported that TGF-β1 is highly expressed in and secreted by cancer stem cells (15, 16), so it is rational to explore if there is a positive loop regulation mechanism exist here; herein, we evaluated TGF-β1 expression in PKH^hi^, PKH^low^, and PKH^neg^ cells by flow cytometry. The results showed that the TGF-β1 expression was highest in PKH^hi^ cells (22.1 ± 4.8%) and decreased as PKH label intensity declined (PKH^low^ 3.3 ± 1.9%; PKH^neg^ 0.8 ± 0.5%, Figure 4A). However, although TGF-β1 protein was upregulated in PKH^hi^ cells, its mRNA levels did not differ significantly among three subsets (Figure S6D). This disparity between protein and mRNA in TGF-β expression suggests that a post-transcriptional mechanism is involved in TGF-β1 regulation.

miRNA-329 was reported to be a tumor suppressor gene that inhibits TGF-β1 (17), and it was interesting to find a relatively low expression level of miR-329 in the PKH^hi^ population (Figure 1B). We then performed RT-PCR to verify the expression of miR-329 in different subsets of cells, and the results confirmed significant, high miR-329 levels in the PKH^low^ and PKH^neg^ populations (Figure 4B), which indicated that the miR-329 level was negatively correlated with the stemness of CCSCs. To confirm that miR-329 regulated the production of TGF-β1, we performed ELISA with specific antibodies. The results showed that cells transfected with the miR-329 mimic increased the levels of both the total and active forms of TGF-β1 (Figure 4C). Afterwards, we determined that the 3‘UTR of TGF-β1 contained highly conserved miR-329 binding sites (Figure 4D), and the luciferase reporter assay found that the activity of a luciferase reporter containing the TGF-β1 3'UTR decreased by 60% upon co-transfection with the miR-329 mimic but increased by 100% upon co-transfection with the anti-miR-329 mimic (Figure S6A). Western blotting also confirmed that the expression of the TGF-β1 could be inhibited by miR-329 mimics (Figure 4E).

We also assessed the impact of miR-329 on the tumor initiation ability and stemness of colon cancer cells. We found that stable expression of the anti-miR-329 mimic potentiated spheroid formation, while overexpression of the miR-329 mimic inhibited sphere formation in both P1 and SW480 cells, which could be rescued by supplement with TGF-β1 (Figure S6B). In addition, in a xenograft tumor model, 1 × 10^4^ cells from each group were injected subcutaneously, and the results revealed that the expression of miR-329 was negatively associated with tumor formation capacity (Figures 4F,G). All these data demonstrated miR-329 played as a tumor suppressive gene and negatively regulated TGF-β pathway in colon cancer stem cells.

### miR-4666-3p and miR-329 Synergistically Regulate the Stemness of CCSCs via the TGF-β/Smad Pathway

**Figure 5:**
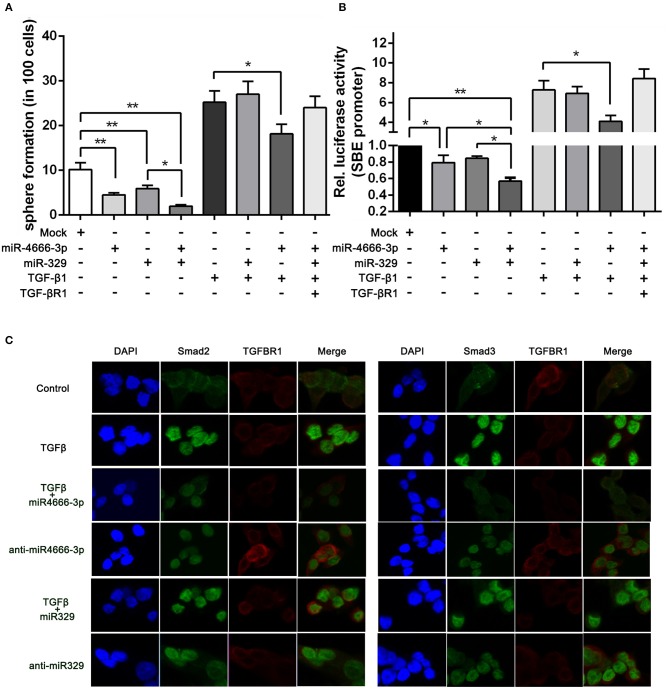
miR-4666-3p and miR-329 regulate the stemness of CCSCs via TGF-β/Smad pathway. **(A)** Sphere formation assay with distinct treatment combinations; **(B)** The activity of the TGF-β/Smad pathway was measured by the SBE luciferase reporter system (presented as P1 cells); **(C)** Immunofluorescence assay to assess the expression of TGFBR1 and nuclear expression of Smad2/3 in treated P1 cells. The data are shown as one typical result from three independent experiments with similar results or as the mean ± SD of three independent experiments. *P < 0.05, **P < 0.01.

Because the above evidence demonstrated that miR-4666-3p and miR-329 both acted as tumor suppressor genes, were expressed at low levels in PKH^hi^ cells and were related to the TGF-β pathway, we tried to determine whether these two microRNAs acted synergistically to affect the stemness and TGF-β/Smad pathway. Since the above data already proved miR-4666-3p and miR-329 can individually suppress the sphere formation and the activity of TGF-β/smad pathway, the simultaneously introduction of both miR-4666-3p and miR-329 showed a cooperative repression function, while addition of 10 ng/ml TGF-β1 or transfected with TGF-βR1 overexpressing plasmid (without its 3′UTR) revealed a rescue effect (Figures 5A,B).

To further identify whether miR-329 and miR-4666-3p impeded activation of TGF-β/Smad pathway, we performed an immunofluorescence assay. The results revealed that TGF-β1 greatly potentiated the translocation of Smad2 and Smad3. This effect could be partially blocked by overexpression of miR-4666-3p, while the translocation of Smad2/3 was increased in the anti-miR-329 stable cell lines (Figure 5C). Besides, western-blot assay revealed anti-miR-4666-3p and anti-miR-329 increased Smad2/3 phosphorylation, and overexpression of miR-4666-3p could partially block the stimulation effect of TGF-β1 (10 ng/ml) on Smad2/3 phosphorylation (Figure S6C). The above results indicated that miR-329 and miR-4666-3p cooperated to regulate the stemness of CCSCs by inhibiting canonical TGF-β/Smad signaling.

### miR-4666-3p and miR-329 Impacts the Clinicopathological Features of CRC

**Figure 6:**
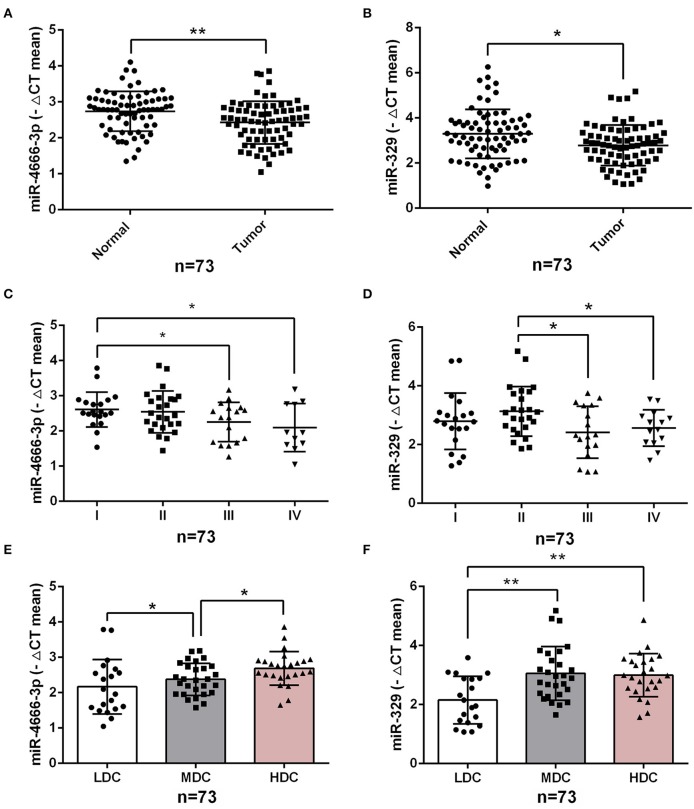
Low expression of miR-4666-3p and miR-329 are related to advanced clinicopathological features. **(A,B)** miR-4666-3p and miR-329 expression assessed by qRT-PCR in 73 pairs of paraffin-embedded CRC and adjacent normal tissue; **(C,D)** miR-4666-3p and miR-329 expression level stratified by different tumor stages; **(E,F)** miR-4666-3p and miR-329 expression level stratified by different tumor grades. LDC, low differentiated cancer; MDC, middle differentiated cancer; HDC, high differentiated cancer. All miR-4666-3p or miR-329 expression levels were presented as ΔCt value = Ct(U6)-Ct(miR-4666-3p or miR-329). Data are represented as the mean ± SD; *P < 0.05, **P < 0.01.

To investigate the impact of miR-4666-3p and miR-329 expression on the clinical features of human CRC, we analyzed our data from 73 CRC patients (Table S1) and found that the expression level of both miR-4666-3p and miR-329 were much higher in normal tissue than in paired tumor specimens (*p* < 0.01, Figures 6A,B). Furthermore, miR-4666-3p and miR-329 expression was negatively correlated with the TNM (tumor, node, metastasis) stage (Figures 6C,D) and histological stage in CRC patients (Figures 6E,F). These findings suggested that miR-4666-3p and miR-329 was negatively associated with tumor invasiveness and progression, and that its expression level might be a prognostic factor in human CRC.

## Discussion

Here, we demonstrate that miR-4666-3p and miR-329 are expressed at low levels in colon cancer stem cells but are upregulated by cell division in non-CCSCs. Furthermore, we find that these two microRNAs work synergistically to inhibit the stemness of CCSCs by targeting the TGF-β/Smad pathway. It has been proven that microRNAs play a pivotal role in maintaining the stemness of CSCs, and some of them could be applied as therapeutic agents in cancer. Previously, we found that cholesterol-conjugated miR-27 mimics could target VEGF-C (Vascular endothelial growth factor C) *in vivo* to inhibit tumor progression and angiogenesis in colorectal cancer (18), and it has also been reported that miR-27 could target the Wnt pathway (19), which is important for stemness maintenance in CCSCs (20). In addition, several tumor suppressor miRNA mimics, such as the let-7 family, miR-34a, miR-143, miR-145, and miR-200 family, are being or have already been developed as therapeutics for clinical trials. It is worth noting that almost all of these miRNAs are also well-known for regulating cancer stem cells (21–23).

Cancer stem cells are considered the root of tumor initiation and metastasis. Abundant evidence suggests that CSCs comprise quiescent CSCs (QCSCs) and rapid cycling, or invasive CSCs, and rapid cycling CSCs can differentiate from QCSCs (2, 24). Here, we isolated quiescent cancer cells with a label staining assay (PKH dye) in serum-free culture medium, as we have previously demonstrated that PKH^hi^ cells strongly exhibit stem cell-like properties (2). We analyzed the microRNA expression profile difference between PKH^hi^ cells and the rest of the cancer cells. To the best of our knowledge, this is the first report screening the differences in expression of microRNAs between QCSCs and non-QCSCs, and by assessing the CRC cell line SW480 and primary CRC cells, we identified 29 differentially regulated miRNAs. We found that miR-4666-3p is the only miRNA downregulated in the PKH^hi^ subpopulation of both SW480 and P1 cells. Here, we prove that miR-4666-3p could target IFN-γR1/2, which explains the higher sensitivity of PKH^hi^ cells to the apoptotic effect of IFN-γ compared to the rest of the cancer cell population (2). Furthermore, we found that upregulation of miR-4666-3p could also suppress stemness gene expression, sphere formation and xenograft generation, but these effects could not be explained as alterations in the expression of IFN-γR (Figures S4A, B). Therefore, we performed bioinformatic analysis to investigate other potential targets that affect the stemness of CCSCs.

It has been reported that TGF-β signaling holds a key position in cancer stem cells. The activation of the TGF-β pathway is involved in regulating EMT (25), promoting invasiveness and metastasis of CCSCs (26), and inducing the secretion of angiogenesis-related factors, such as fibroblast growth factor 2 and vascular endothelial growth factor, by CCSCs (25, 27). TGF-β signaling is transduced by TGF-β receptors, which include TGF-βR1, 2, and 3. Studies have revealed that TGFBRs' expression is closely associated with cancer stem cells. Yusra et al. (28) found that the expression of TGFBR1 and TGFBR3 was higher in CD133-positive colon cancer stem cells, suggesting that CD133+ CCSCs have an increased sensitivity to TGF-β. The gene expression profile of mammary cancer stem-like cells revealed high expression of TGFBR2 (29). TGFBR1 was also reported as a therapeutic target to suppress stem-like features of these cells (12, 30). As we proved here by several methods, TGFBR1 is a functional downstream target of miR-4666-3p, and alterations in miR-4666-3p and TGFBR1 expression could affect the stemness of colon cancer cells and activation of TGF-β/Smad pathway.

**Figure 7:**
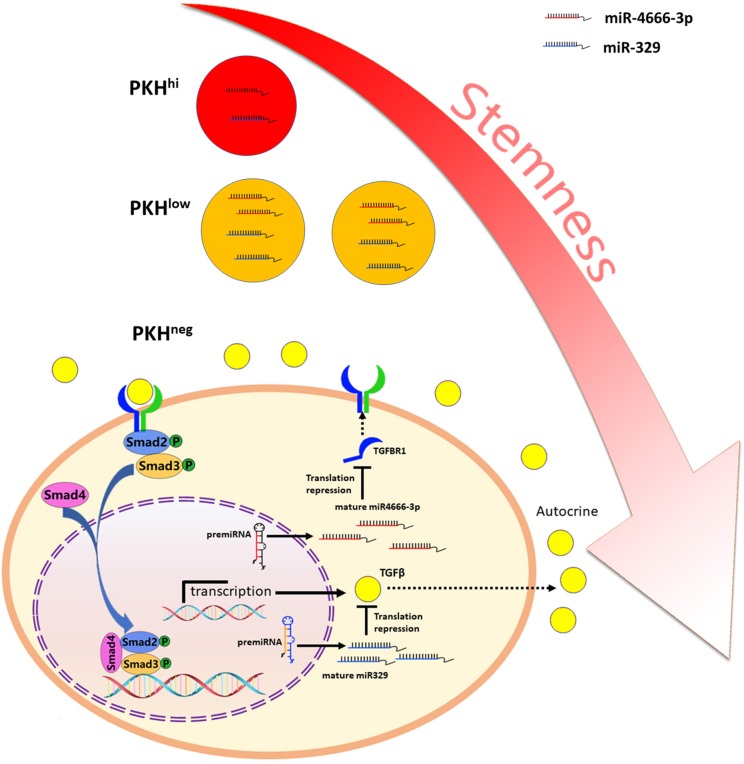
Schematic illustration of miR-4666-3p and miR-329 are both low expressed in non-QCCSCs and synergistically regulate TGF-β/Smad pathway.

Besides, it is noted that autocrine of TGF-β1 by cancer cells play important part in regulation of TGF-β/Smad pathway. Abundant evidence has highlighted the importance of the TGF-β autocrine mechanism in CSCs. Liu et al. (31) reported that TGF-β autocrine signaling greatly enhanced the ability of cells to grow anchorage independently in serum-free medium and potentiated the stemness and invasiveness of CSCs. Yeh et al. (32) found that elevated expression of PSPC1 (paraspeckle component 1) potentiated the TGF-β1 autocrine pathway and activated the TGF-β/Smad pathway, which promoted stemness, EMT and invasiveness in various cancer cells. Here, we found that PKH^hi^ cells produce higher levels of TGF-β1 than PKH^low^ and PKH^neg^ cells, which could be attributed to the low miR-329 expression level in PKH^hi^ cells. miR-329 has been reported as a tumor suppressor gene and is related to favorable clinicopathological features in various cancers (17, 33, 34). TGF-β1 has been revealed to be a target of miR-329 (17), and here we also identified it as a functional target of miR-329. In addition, transfection of miR-329 mimics greatly inhibited the spheroid and xenograft formation abilities of colorectal cancer cells. Furthermore, our luciferase and immunofluorescence assays showed that miR-329 and miR-4666-3p mimics synergistically inhibit the spheroid formation and activation of TGF-β/Smad signaling. Here, although miR-4666-3p and miR-329 target on different gene, here we demonstrated both expressed at low levels in CCSCs and cooperated on the same signaling pathway (Figure 7).

However, the dysregulated expression of miRNAs in cancer cells is still not fully understood, and several studies indicate that underlying causes may contribute to the aberrant regulation of specific pathways. For example, activation of the MAPK and NF-κB (nuclear factor kappa-B) pathways could upregulate the transcriptional factor C/EBPβ, thereby increasing the expression of let-7f and miR-146 (35, 36). Here, we acknowledge that the mechanism behind the increase in the expression levels of miR-4666-3p and miR-329 that occurs during stem cell division is still undetermined, and future studies are needed to elucidate the detailed mechanism.

## Conclusion

To our knowledge, this is the first study to report this specific and novel mechanism by which miRNAs cooperate to suppress the TGF-β/Smad pathway in colorectal cancer. Our results suggest that targeting multiple miRNAs in a single therapy could be more comprehensive and effective for cancer treatment.

## Data Availability Statement

All datasets generated for this study are included in the article/Supplementary Material.

## Ethics Statement

This study was approved by the Ethical Committee of the Zhejiang Provincial People's hospital, Hangzhou Medical College. The patients were interviewed prior to inclusion in the study and were provided with written and verbal information regarding the study. All patients provided written informed consent. All methods were performed in accordance with the relevant guidelines and regulations. This study was approved by the ethics committee of the Laboratory Animal Research Center of Zhejiang Medical Academy.

## Author Contributions

CN and JH conceived and designed the study. JY, YS, XH, HY, and QX performed experiment and data collecting. JY, QF, JL, and WX analyzed data and performed the statistical analysis. CN critically revised the manuscript. All authors commented on drafts of the paper and approved the final manuscript.

### Conflict of Interest

The authors declare that the research was conducted in the absence of any commercial or financial relationships that could be construed as a potential conflict of interest.

## Abbreviations

CCSC: colorectal cancer stem cells
CSC: cancer stem cell
CRC: colorectal cancer
ELISA: enzyme-linked immunosorbent assays
FCM: flow cytometry
FACS: fluorescence activated Cell Sorting
IFNGR: Interferon gamma receptor
NF-κB: nuclear factor kappa-B
MAPK: mitogen-activated protein kinase
PSPC1: paraspeckle component 1
KEGG: Kyoto Encyclopedia of Genes and Genomes
QCCSCs: quiescent cancer stem cells
TCGA: The Cancer Genome Atlas
TNM: tumor, node, metastasis
VEGF-C: Vascular endothelial growth factor C.
