# Supplementary material for: miR-4666-3p and miR-329 Synergistically Suppress the Stemness of Colorectal Cancer Cells via Targeting TGF-β/Smad Pathway
Source: Front Oncol. 2019 Nov 19;9:1251. doi: 10.3389/fonc.2019.01251 (PMC6880832; doi:10.3389/fonc.2019.01251)
Supplement: Supplementary file 1 [file Table_1.DOCX]

**Figure S1**


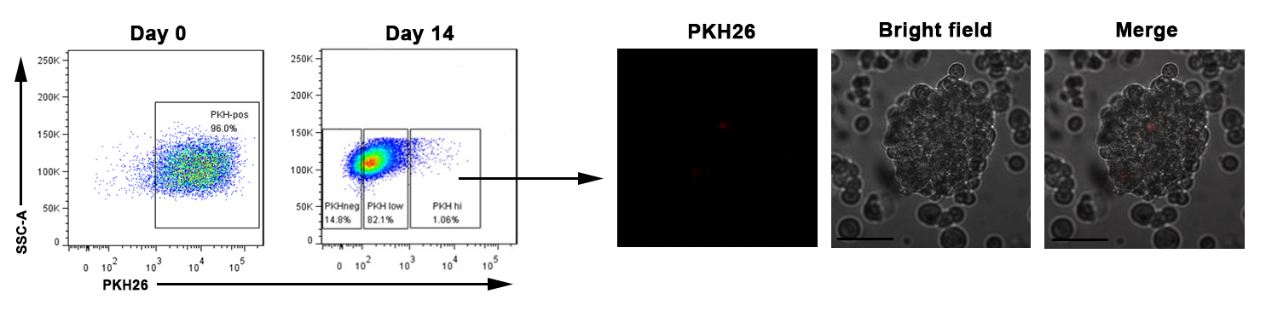


**Figure S1** The sorting strategy was presented as P1 cells. PKH labeled P1 cancer cells were seeded in SF culture conditions, and PKH intensity was detected by FCM on days 0 and 14, colon sphere on day 14 was derived from PKH26-labeled cells. Scale bar = 100 μm.

**Figure S2**


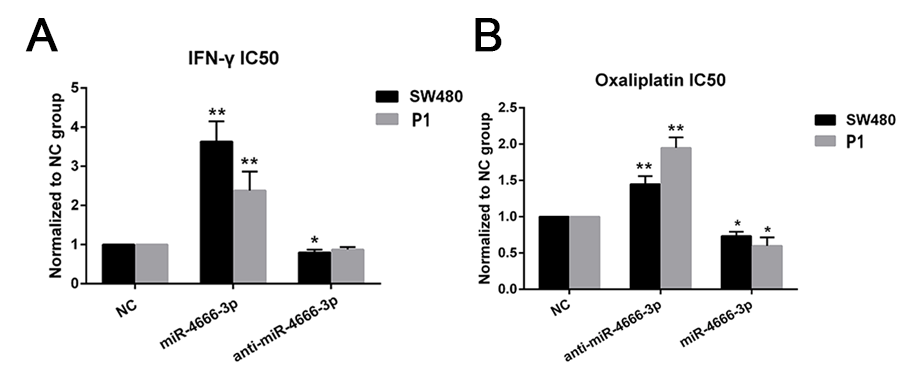


**Figure S2** IC50 of IFN-γ (A) and Oxaliplatin (B) was assessed by MTS in different cell subsets. The data are shown as one typical result from 3 independent experiments with similar results or as the mean ± SD of 3 independent experiments. vs NC group, **P* < .05, ***P* < .01.

**Figure S3**


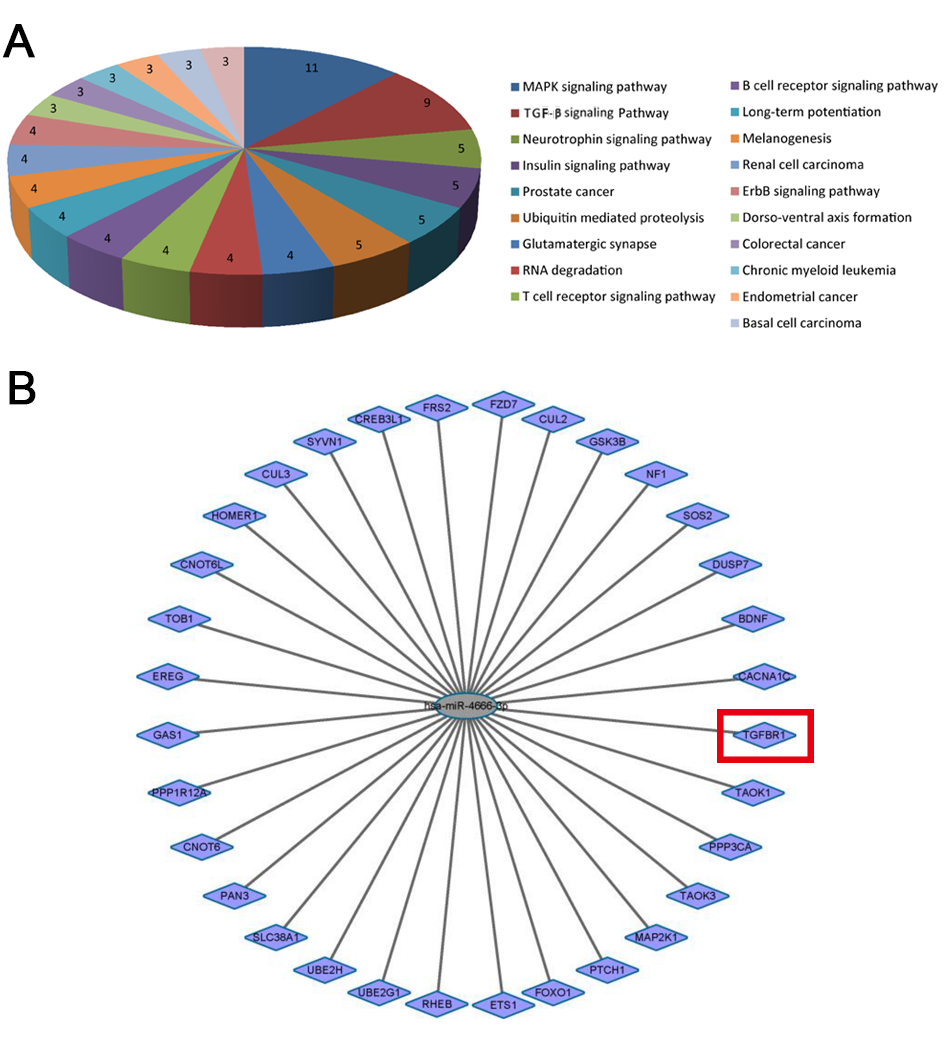


**Figure S3** Bioinformatic analysis of miR-4666-3p target gene. (A) KEGG (Kyoto Encyclopedia of Genes and Genomes) analysis results presented as a pie graph; (B) Network diagram presents 33 targeted genes retrieved from the KEGG analysis.

**Figure S4**


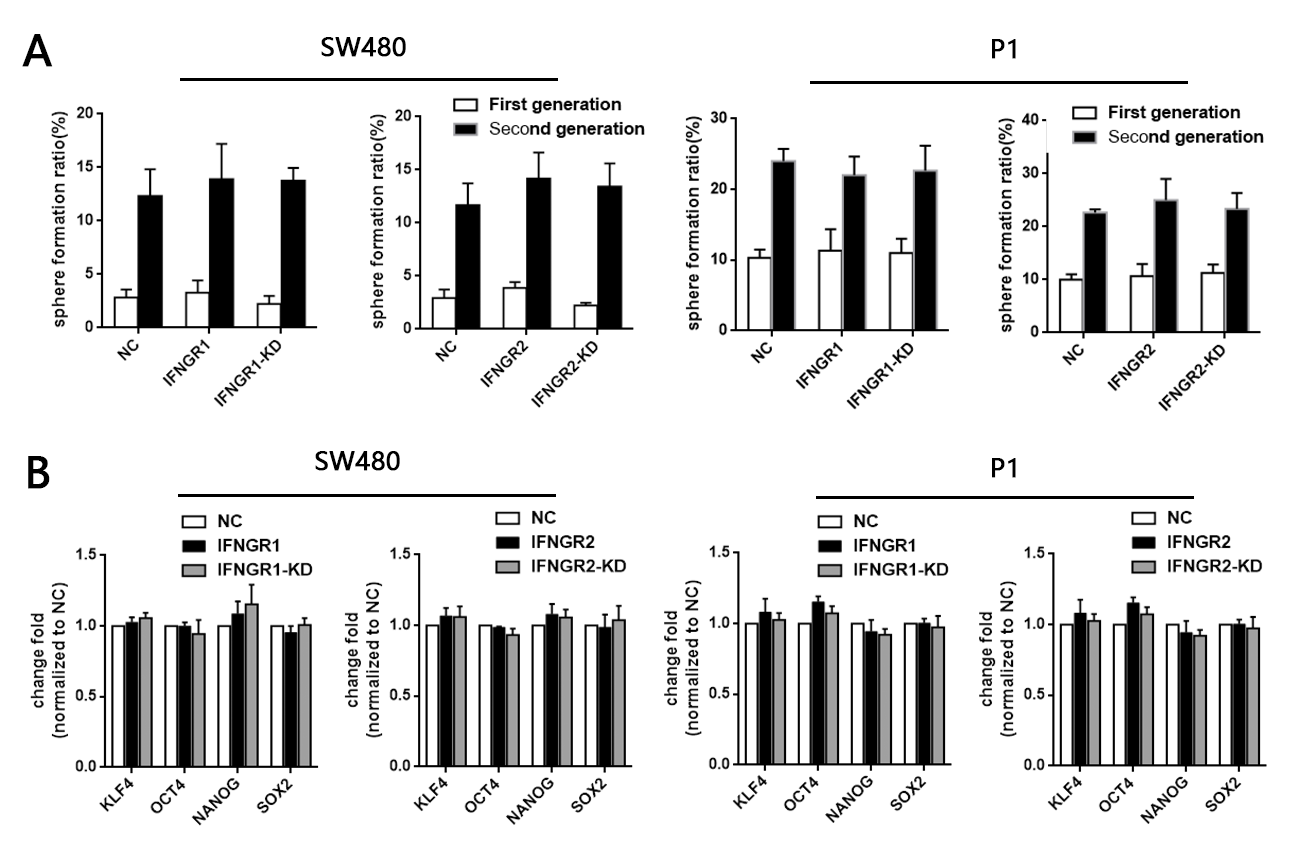


**Figure S4** Evaluate the affection of IFN-γR1/2 expression on stemness on colorectal cancer cells. (A) Serial sphere formation assay with control, IFN-γR1 and IFN-γR2 high- and low-stable expressing SW480 and P1 cells; (B) The expression of stemness genes was assessed by qRT-PCR in control, IFN-γR1 and IFN-γR2 high- and low-stable expressing SW480 and P1 cells. The data are shown as the mean ± SD of 3 independent experiments.

**Figure S5**


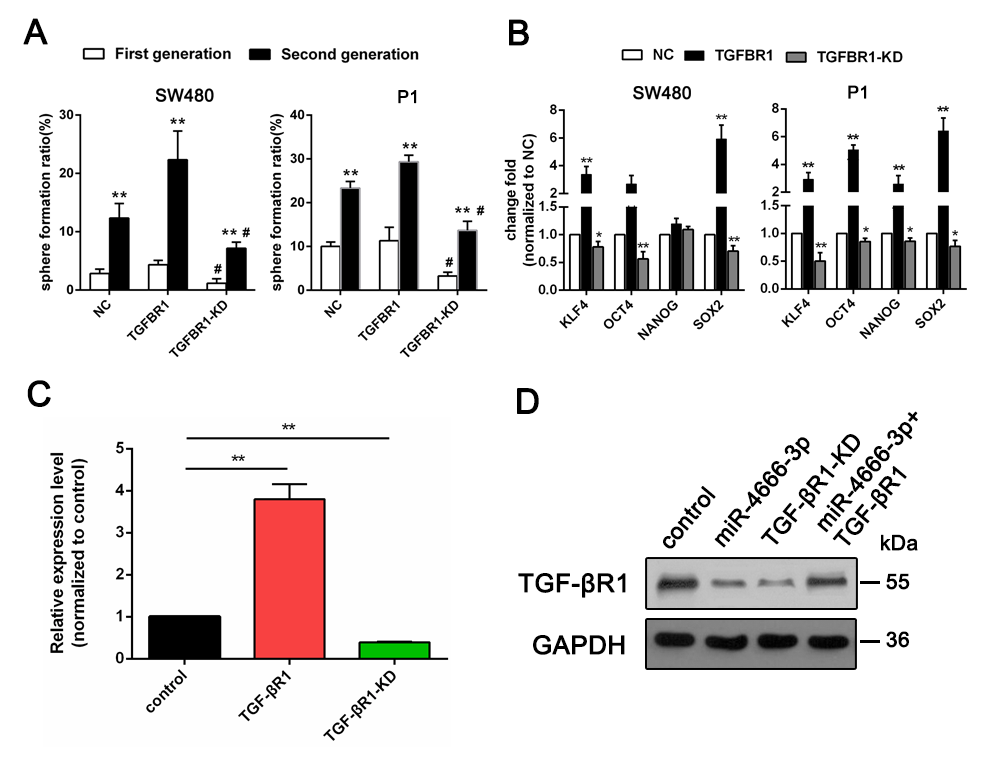


**Figure S5** Evaluate the affection of TGFBR1 expression on stemness on colorectal cancer cells. (A) Serial sphere formation with control, TGFBR1 high- and TGFBR1 low-expressing CRC cells (* vs first generation; # vs NC group); (B) The expression of stemness genes was assessed by qRT-PCR in control, TGFBR1 high- and TGFRB1 low-expressing CRC cells.; (C-D) qRT-PCR and western-blot validate the mRNA and protein expression in TGF-βR1 knockdown or overexpression stable P1 cells. The data are shown as one typical result from 3 independent experiments with similar results or as the mean ± SD of 3 independent experiments. *,#*P* < .05, **,##*P* < .01.

**Figure S6**


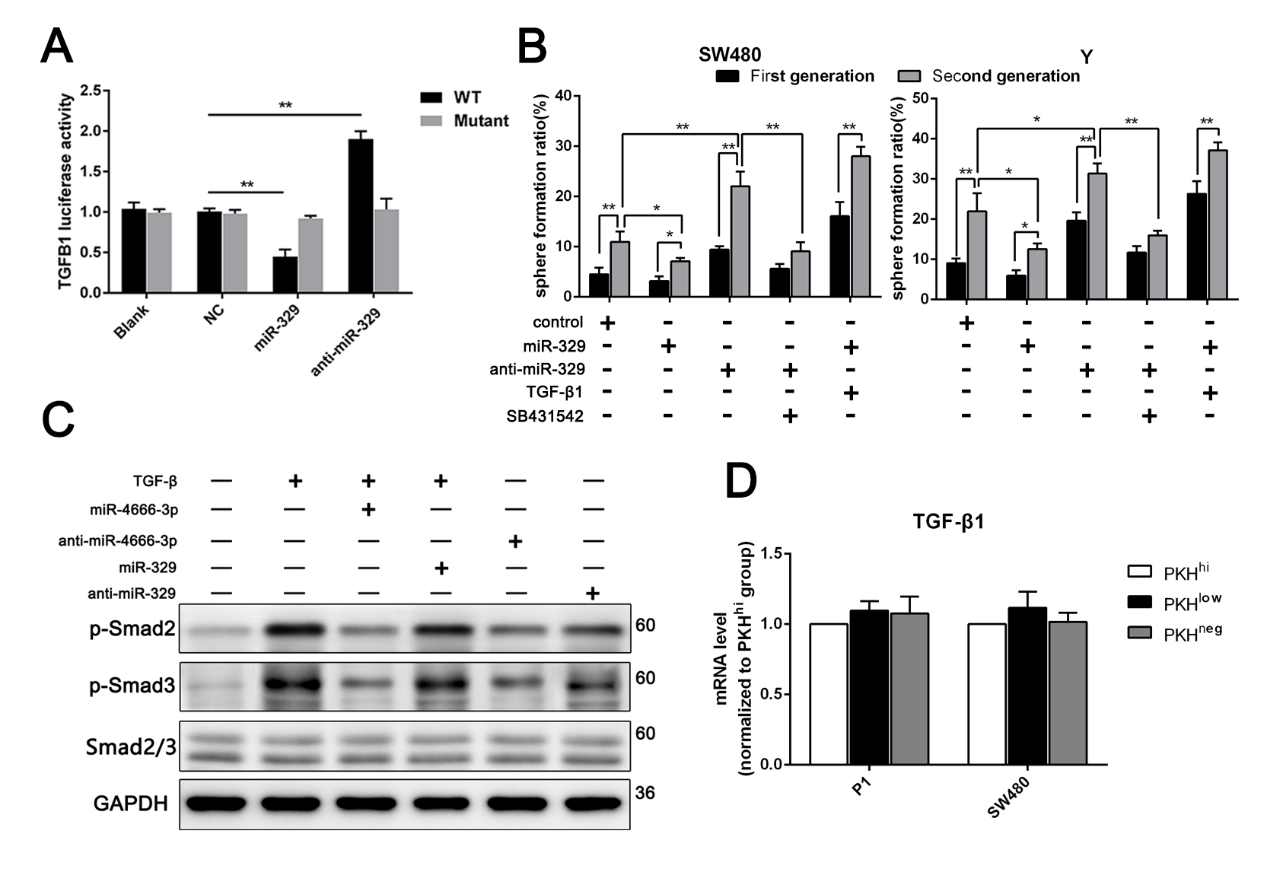


**Figure S6** Identify the effect of miR-329 and miR4666-3p on TGF-β/SMAD pathway，andTGF-β1expssion in PKH^hi/low/neg^ cells. (A) 293T cells were cotransfected with empty pmirGLO Dual-Luciferase reporter plasmids or TGFβ1 3’UTR firefly luciferase reporter plasmids, pRL-TK-luciferase plasmids, and the miR-329 or anti-miR-329 mimic. After 48 h, firefly luciferase activity was measured and normalized to that of Renilla luciferase; (B) Sphere formation assay of CRC cells with distinct treatment combinations ; (C) Smad2 and Smad3 phosphorylation were detected by western-blot with distinct treatment combinations in P1 cells; (D) mRNA levels of TGF-β1 in PKHhi/low/neg subpopulations were assessed by RT-PCR. The data are shown as one typical result from 3 independent experiments with similar results or as the mean ± SD of 3 independent experiments. **P* < .05, ***P* < .01.

**Table S1** **Clinicopathological features of CRC patients.**

| Characteristics | Number | |
| --- | --- | --- |
| Gender |  | |
| Male | 35 | |
| Female | 38 | |
| Age (years) |  | |
| Male | 59 (26–79) | |
| Female | 63 (41–81) | |
| Primary Site |  | |
| Rectum | 4 | |
| Sigmoid colon | 25 | |
| Right hemicolon | 23 | |
| Left hemicolon | 21 | |
| Pathological Type |  | |
| Tubular adenocarcinoma | 32 | |
| Papillary adenocarcinoma | 34 | |
| Mucinous adenocarcinoma | 4 | |
| Complex | 3 | |
| Differentiation |  | |
| Well | 20 | |
| Moderate | 28 | |
| Poor | 25 | |
| Invasion depth |  | |
| T1 | 13 | |
| T2 | 19 | |
| T3 | 23 | |
| T4 | 12 | |
| Lymph node metastasis |  | |
| N0 | 14 | |
| N1 | 24 | |
| N2 | 35 | |
| Metastasis | | |
| Yes | | 11 |
| No | | 62 |
| TNM stage |  | |
| I | 20 | |
| II | 25 | |
| III | 17 | |
| IV | 11 | |

**Supplementary methods**

1. PKH 26 staining and isolation

The cells were stained with PKH26, the expression of PKH26 is divided equally among the daughter cells subsequent to each cell cycle. Cancer cells were then sorted on the basis of their different fluorescent intensities, which were then classified as the PKH^hi^, PKH^low^ and PKH^neg^. PKH^hi.^ cells were identified as the subpopulation whose fluorescence intensity as strong as initial staining with PKH26, PKH^neg^ cells were gated based on the cells had not stained with PKH26, and the intermediate part defined as PKH^low^ subpopulation.

1. ELISA

The total and active TGF-β1 concentrations were determined with a Total TGF-β1 Precoated ELISA Kit and a Free Active TGF-β1 Precoated ELISA Kit (Biolegend, San Diego, USA), respectively. The procedure was performed according to the manufacturer’s protocol.

1. Plasmid construction

The 3’UTR of IFNGR1/2 and TGFBR1 containing the miR-4666-3p target site (5’-AACTCATTGTATTCAATA-3’) or a mutant 3’UTR of IFNGR1/2 and TGFBR1 (5’-AACTCGACACGTTCAATA-3’) and the 3’UTR of TGFβ1 containing the miR-329 target site (5’-AACTCGGTGTGTTCAATA-3’) or a mutant 3’UTR of TGFβ1 (5’-AACTC GACACGTTCAATA-3’) were cloned into the SacI/XhoI site of the pmirGLO Dual-Luciferase miRNA Target Expression Vector (Promega).

1. Establishment of miR-4666-3p/miR-329-overexpressing and anti-miR-4666-3p/anti-miR-329-expressing stable cell lines

The miR-4666-3p/miR-329 or anti-miR-4666-3p/anti-miR-329 lentiviral particles (PG-LV3-H1-miR-4666-3p-Puro/PG-LV3-H1-miR-4666-3p inhibitor-Puro; PG-LV3-H1-miR-329-Puro/PG-LV3-H1-miR-329 inhibitor-Puro) (GenePharma Tech, Shanghai, China) were transfected into SW480 or P1 cells. Cells were selected with 5 μg/ml puromycin (Sangon Biotech) 48 h after transfection. The efficiency of overexpression or downregulation was verified by RT-PCR.

1. Establishment of IFNGR1/2, TGF-βR1 knockdown or overexpression stable cells

IFNγR1, IFN-γR2 and TGF-βR1 overexpression or shRNA lentiviral plasmid (GenePharma Tech, Shanghai, China) was transfected into SW480 or P1 cells using Lipofectamine 2000 (Invitrogen) in accordance with the manufacturer’s instructions. Cells were selected with 5 μg/ml puromycin 48 h after transfection and the knockdown efficiency verified by RT-PCR.

1. Determination of the half maximal inhibitory concentration (IC50) with MTS

To determine the half-maximal inhibitory concentrations (IC50) of IFN-γ and oxaliplatin in SW480 and P1 cells, cells were seeded into 96-well plates and treated with IFN-γ or oxaliplatin for 24h, and the viable cells were evaluated with the CellTiter 96® Aqueous Non-Radioactive Cell Proliferation Assay (MTS, Promega). All experimental procedures followed the manufacturer’s instructions. IC50 values were determined by plotting a linear regression curve.
