# Supplementary figures and images for: miR-4666-3p and miR-329 Synergistically Suppress the Stemness of Colorectal Cancer Cells via Targeting TGF-β/Smad Pathway
Source: Front Oncol. 2019 Nov 19;9:1251. doi: 10.3389/fonc.2019.01251 (PMC6880832; doi:10.3389/fonc.2019.01251)

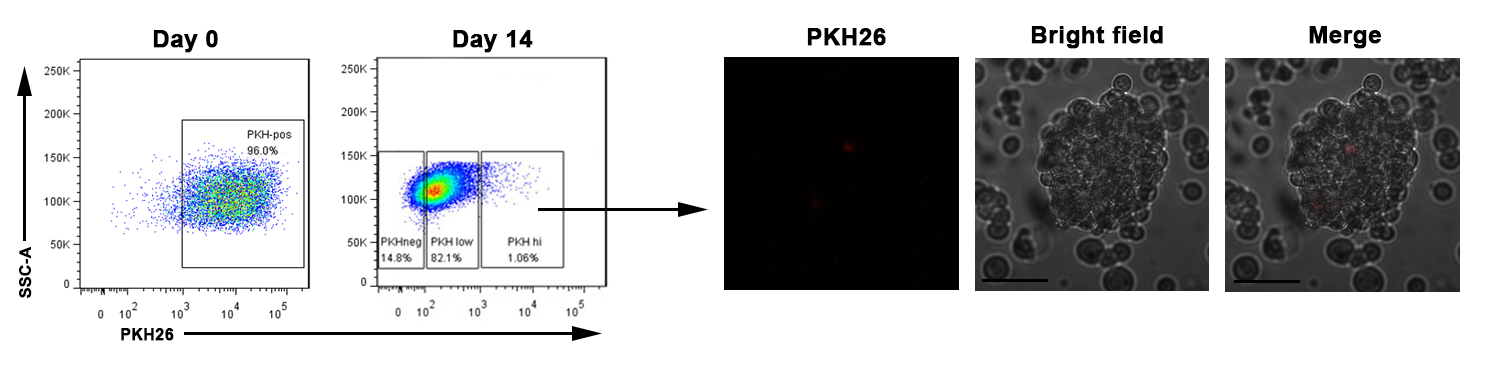

Supplement: Supplementary file 2 [file Data_Sheet_1.ZIP › Fig S1.tif]

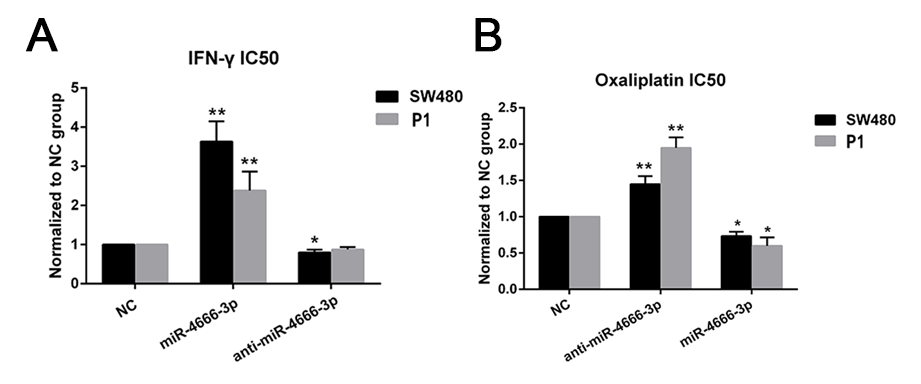

Supplement: Supplementary file 2 [file Data_Sheet_1.ZIP › Fig S2.tif]

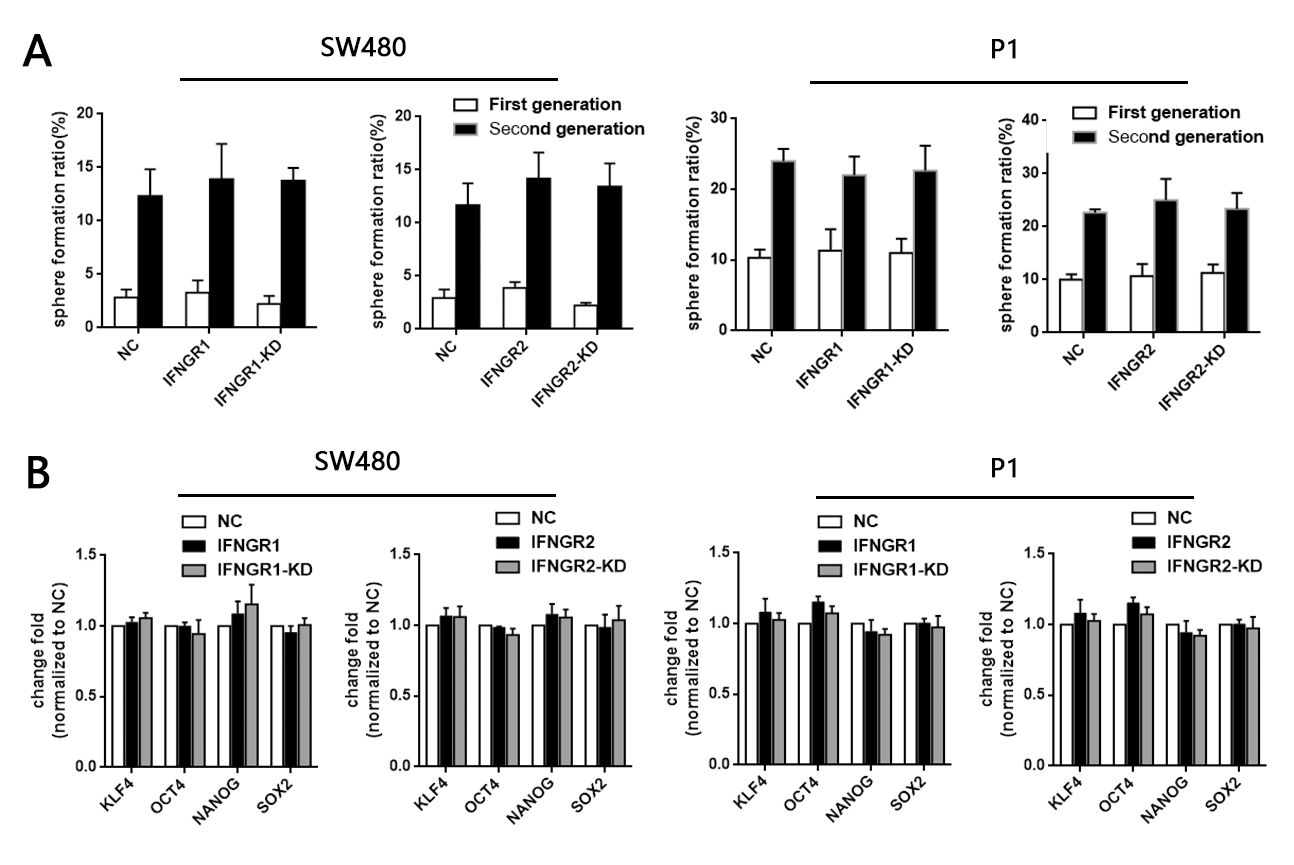

Supplement: Supplementary file 2 [file Data_Sheet_1.ZIP › Fig S3.tif]

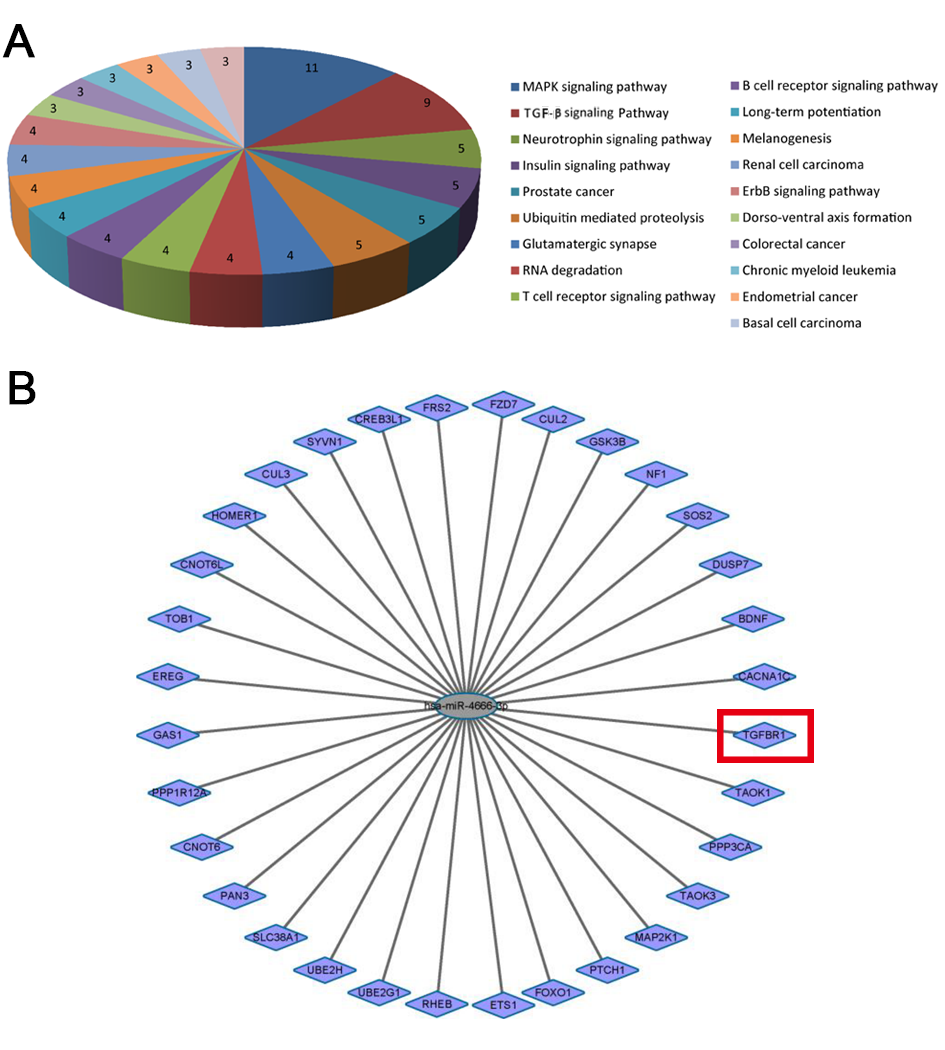

Supplement: Supplementary file 2 [file Data_Sheet_1.ZIP › Fig S4.tif]

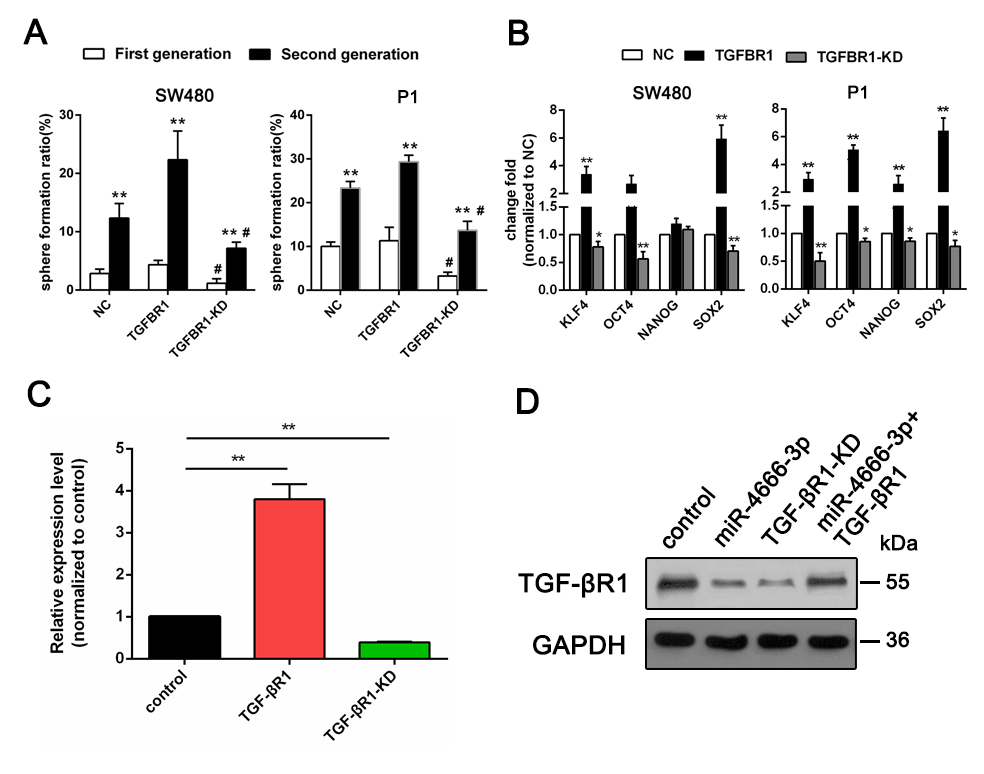

Supplement: Supplementary file 2 [file Data_Sheet_1.ZIP › Fig S5.tif]

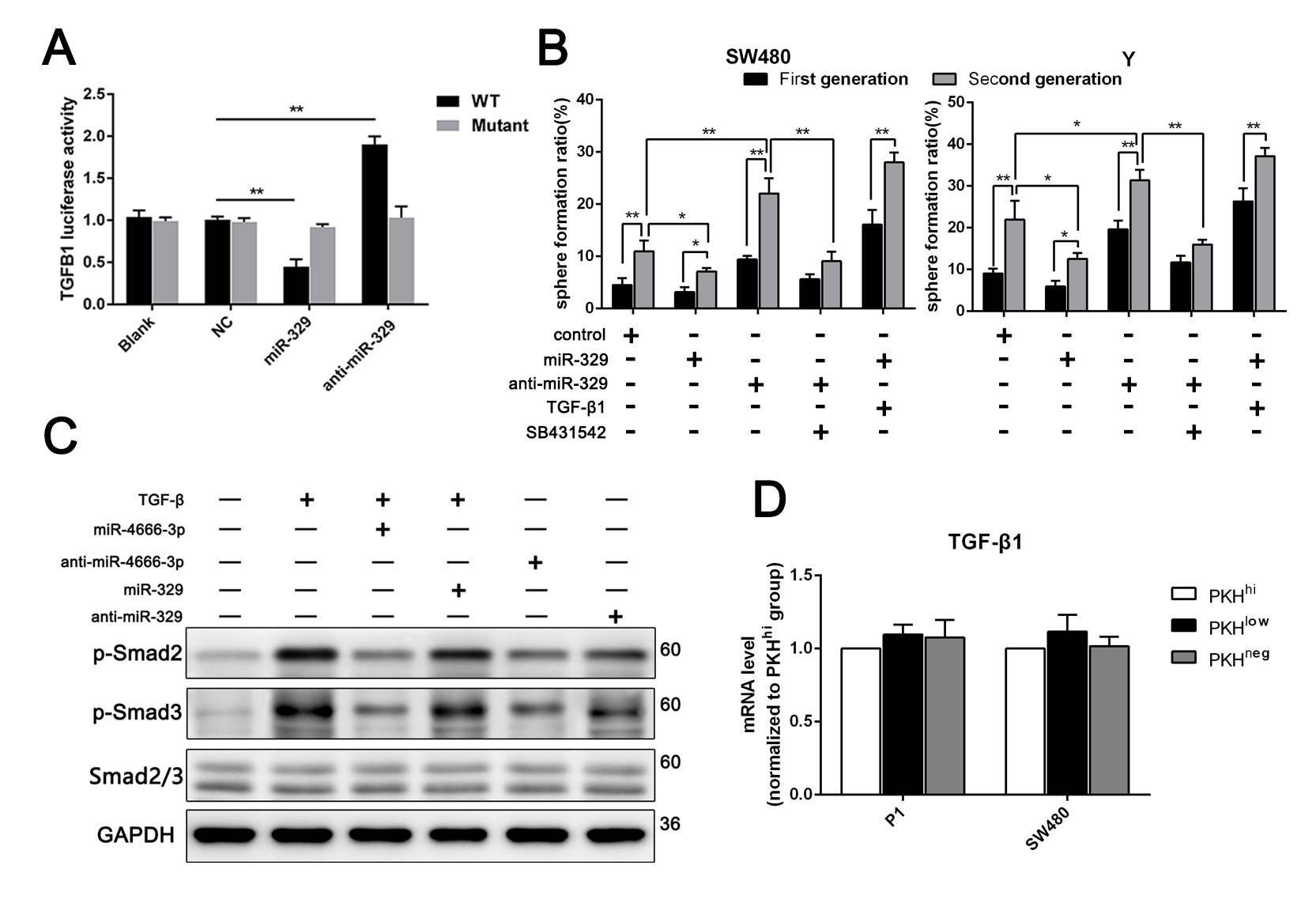

Supplement: Supplementary file 2 [file Data_Sheet_1.ZIP › Fig S6.tif]
